# Biological control needs evolutionary perspectives of ecological interactions

**DOI:** 10.1111/eva.13457

**Published:** 2022-11-01

**Authors:** Arnaud Sentis, Jean‐Louis Hemptinne, Alexandra Magro, Yannick Outreman

**Affiliations:** ^1^ INRAE Aix Marseille University, UMR RECOVER Aix‐en‐Provence France; ^2^ Laboratoire Évolution et Diversité biologique UMR 5174 CNRS/UPS/IRD Toulouse France; ^3^ Université Fédérale de Toulouse Midi‐Pyrénées – ENSFEA Castanet‐Tolosan France; ^4^ IGEPP, INRAE, Institut Agro Univ Rennes 1 Rennes France

**Keywords:** agents, climate change, evolution, genetics, invasions, pests, symbionts

## Abstract

While ecological interactions have been identified as determinant for biological control efficiency, the role of evolution remains largely underestimated in biological control programs. With the restrictions on the use of both pesticides and exotic biological control agents (BCAs), the evolutionary optimization of local BCAs becomes central for improving the efficiency and the resilience of biological control. In particular, we need to better account for the natural processes of evolution to fully understand the interactions of pests and BCAs, including in biocontrol strategies integrating human manipulations of evolution (i.e., artificial selection and genetic engineering). In agroecosystems, the evolution of BCAs traits and performance depends on heritable phenotypic variation, trait genetic architecture, selection strength, stochastic processes, and other selective forces. Humans can manipulate these natural processes to increase the likelihood of evolutionary trait improvement, by artificially increasing heritable phenotypic variation, strengthening selection, controlling stochastic processes, or overpassing evolution through genetic engineering. We highlight these facets by reviewing recent studies addressing the importance of natural processes of evolution and human manipulations of these processes in biological control. We then discuss the interactions between the natural processes of evolution occurring in agroecosystems and affecting the artificially improved BCAs after their release. We emphasize that biological control cannot be summarized by interactions between species pairs because pests and biological control agents are entangled in diverse communities and are exposed to a multitude of deterministic and stochastic selective forces that can change rapidly in direction and intensity. We conclude that the combination of different evolutionary approaches can help optimize BCAs to remain efficient under changing environmental conditions and, ultimately, favor agroecosystem sustainability.

## 1 INTRODUCTION

Agriculture and food systems are responsible for at least 30% of GHG emissions and are important drivers of the rapid erosion of biodiversity (Díaz et al., 2019; Sun et al., 2022). They must thus rapidly become more sustainable. Pesticides need to be severely restricted, and greater emphasis should be placed on agroecosystem redesign to reduce pest outbreaks and favor local biodiversity, including native natural enemies of pests (Tscharntke et al., 2021). Because arthropod herbivorous pests account for ca. 20% of agricultural production losses worldwide (van Lenteren et al., 2018), biological control (i.e., “The use of living organisms to suppress the population density or impact of a specific pest organism, making it less abundant or less damaging than it would otherwise be,” Eilenberg et al., 2001) offers a promising solution to achieve resilience and sustainability in agricultural systems.

Historically, biological control was strongly improved by an increasing understanding of the trophic interactions between a targeted pest and its natural enemies (DeBach & Rosen, 1974; Huffaker et al., 1976). Over the last decades, the emphasis has been placed on the influence of indirect ecological interactions on the outcome of direct interactions between pests and their enemies (Heimpel & Mills, 2017; Holt & Hochberg, 2001; Rosenheim et al., 1995). The study of these ecological processes remains an important field of research and has yielded significant progresses in classical (i.e., importing, and releasing for establishment, natural enemies to control introduced or native pests), augmentative (i.e., the supplemental release of natural enemies), and conservation (i.e., the use of methods supporting populations of natural enemies present in the agroecosystem and promoting their effectiveness) biological control programs (Heimpel & Mills, 2017). However, development of biocontrol methods has often neglected the fundamental concepts of evolutionary ecology. This is surprising as both evolutionary concepts and methods permeated agriculture for centuries for the domestication of plants and animals or to improve traits of interest. One explanation might be that ecology and evolution have been mainly viewed separately, with evolution considered as a slow process with little influence on the short‐term ecological dynamics that were associated with biocontrol success. Nevertheless, evolution can be fast, resulting in eco‐evolutionary loops (Faillace et al., 2021; Fussmann et al., 2007; Schoener, 2011), and the evolutionary history of a given species or population can have a strong impact on interspecific interactions and biomass distribution across trophic levels (Gil et al., 2004; Sentis et al., 2020). Moreover, in agroecosystems, the importance of rapid pest evolutionary responses to anthropogenic selective pressures has been well documented with the evolution of insect pest resistance to selection imposed by insecticides (i.e., over 500 insect species have developed resistance to one or more insecticides, Whalon et al., 2008). Furthermore, rapid micro‐evolutionary changes have been observed in a number of plant–insect herbivore, pathogen–plant, and arthropod predator–prey interactions (Carroll & Fox, 2008). These evolutionary changes can have important consequences for the regulation of pest populations. For instance, Harmon et al. (2009) investigated both ecological and evolutionary responses of the pea aphid (*Acyrthosiphon pisum*) to increased frequency of heat waves. They documented the rapid evolution of aphid strains following heat waves, which led to a lower aphid control by their ladybeetle predators. In a recent study, Sentis et al. (2020) investigated how intraspecific differences among pea aphid lineages specialized on different host‐plants influence trophic cascade strength in a ladybeetle‐aphid‐plant system. They found that the occurrence and strength of the trophic cascade are strongly determined by aphids' lineage and host‐plant specialization. Altogether, these studies indicate that evolution should not be underestimated as a driving force influencing biological control efficiency.

Here, we review recent findings highlighting the value of an evolutionary perspective for biological control and identify how they could be more thoroughly considered. We focus on the evolution of phenotypic traits in biological control agents (BCAs), considering both micro‐BCAs (viruses, bacteria, and fungi) and macro‐BCAs (parasitoids, predators, and nematodes), with a stronger focus on studies concerning macro‐BCAs. Although the role of evolution in the context of biocontrol has been reviewed recently (Kruitwagen et al., 2018; Leung et al., 2020; Lirakis & Magalhães, 2019; Lommen et al., 2017), we propose a new perspective on the different facets of evolutionary biological control by highlighting the natural processes of evolution (NPE) and how they can be manipulated by humans to increase the likelihood of evolutionary increased performance. In agroecosystems, the evolution of BCA traits and performance depends on heritable phenotypic variation, trait genetic architecture, selection strength, stochastic processes, and other selective forces (Table 1). Humans can interfere with these natural processes to direct evolution and increase the chance and the magnitude of evolution to occur in the right direction for biological control. Human manipulations of evolution (HME) include here all evolutionary and engineering methods aiming at improving a particular trait of interest (e.g., artificial selection and genome editing). In most cases, human manipulations of evolution must account for the natural processes that can foster or hamper evolution. For instance, evolution by artificial (or natural) selection requires the presence of additive genetic variation in the population, but the selection process itself leads to a depletion of this genetic variation. Additionally, genetic trade‐offs among traits can hinder HME. Although HME can rapidly improve BCA traits, BCA performance must be tested in the field as there is a risk of not being adapted to the recipient environment (Table 1). We highlight these different facets of evolutionary biological control by first reviewing the potential of HME to improve traits of interest for biological control. We then discuss the NPE in agroecosystems and (i) the application of evolutionary approaches for the performance of BCAs under climate change, (ii) the importance of evolution following the release of BCAs in the environment, (iii) the evolution of intra and interspecific interactions within guild of natural enemies, and (iv) the evolutionary importance of endosymbiotic microorganisms harbored by the interacting species. We finally discuss the importance of considering the interplay between HME and NPE for optimizing biological control.

**TABLE 1:** Summary of the natural processes of evolution and how humans can manipulate these processes to increase BCA performance through evolution.

	Evolution and biological control	
	Natural processes of evolution (NPE)	Human manipulations of evolution (HME)
Sources of heritable phenotypic variation	Standing genetic variation, spontaneous mutations, epigenetic variation, heritable microbial symbionts	Artificially increased by induced mutations, genetic engineering, or increased standing genetic variation (e.g., mixing source populations)
Selective forces	Multiple selective forces that depend on local biodiversity and environmental conditions	Often a single directional selective force established by humans
Evolutionary processes	Natural selection, gene flow, stochastic processes (genetic, environmental and demographic stochasticities; genetic drift)	Selection strength is artificially increased Stochastic processes strongly depend on the experimental design. They can be minimized by increasing population size or diversity and by controlling the environment Evolution can be overpassed by genetic engineering (GMO, genome edition)
Effect on trait of interest	Positive, neutral, or negative as forces can be diffuse and unpredictable Depends on genetic architecture of traits	Often positive because of human directional manipulations; can also be neutral, in case of, for example, trade‐offs among traits. Depends on genetic architecture of traits

*Note*: In agroecosystems, BCAs and their pests are exposed to multiple selective forces that may improve BCA performance depending on heritable phenotypic variation, selection strength, stochastic processes, and trait genetic architecture. Humans can manipulate these natural processes to increase the likelihood of evolutionary trait improvement by artificially increasing heritable phenotypic variation, strengthening selection, controlling stochastic processes, or overpassing evolution through genetic engineering. Except for genetic engineering, human manipulations must account for the natural processes that can foster or hamper evolution. For instance, genetic drift can hamper evolution by artificial (or natural) selection as the amount of genetic diversity is likely to decrease after several generations of strong selection. Although human manipulations can rapidly improve BCA traits, BCA performance must be tested in the field as there is a risk of not being adapted to the release environment.

## 2 HUMAN MANIPULATIONS OF EVOLUTION: GENETIC AND EVOLUTIONARY APPROACHES TO IMPROVE BIOLOGICAL CONTROL AGENTS

Since the early times of agriculture, humans have selected plants and animals based on intraspecific phenotypic variation to improve traits of plants and animals such as grain size, drought resistance or milk production. In contrast, biological control has been little influenced by genetic applications to improve its efficiency. However, several recent studies reviewed and highlighted the need for integrating genetics and genomics in biological control (Leung et al., 2020; Montserrat et al., 2021). This may offer an avenue for optimizing currently registered BCAs, which is much needed as importation of exotic BCAs is becoming more restricted and, with rapid environmental changes, BCAs currently mass‐reared by factories may no longer be adapted to local conditions. Different evolutionary and genetic approaches are used to optimize phenotypic traits that are important for biological control. Here, we briefly review these different approaches.

Except for genome editing and genetically modified organisms (see below), the main approaches for HME consist in exploiting intraspecific variation to improve phenotypic traits of interest for biological control, such as the ability to control pest populations, thermal tolerance or reduced sensitivity to intraspecific competition, for improving mass production and storage (Bielza et al., 2020; Dumont et al., 2019; Kruitwagen et al., 2018; Lommen et al., 2017; Zhang et al., 2018). A trait can be improved by selection if it has significant genetic intraspecific variation. Nevertheless, the amount of genetic variation for biological control traits is currently poorly investigated and remains largely unknown (Ferguson, 2020; Lirakis & Magalhães, 2019; Wajnberg, 2004). Only 2.4% of the studies investigating genetics of biocontrol traits reported information on genetic variation (e.g., Postic et al., 2021). Characterizing the genetic architecture of biocontrol traits is important because the success of artificial selection often depends on the number and location of the loci involved, and their interactions such as dominance, epistasis, and pleiotropy (Leung et al., 2020). These different data can inform about the genetic architecture of a trait and gene expression along environmental gradients (e.g., temperature and PH), which can then be used to fine‐tune the genetic approach for trait selection and biocontrol optimization (Leung et al., 2020; Montserrat et al., 2021).

Artificial selection or experimental evolution can be used on a wide range of traits such as pesticide resistance or thermal tolerance (reviewed in Lirakis & Magalhães, 2019). Although often successful in improving the targeted trait, artificial selection is rarely implemented in biological control (Lirakis & Magalhães, 2019). The decreasing cost of phenotyping and sequencing makes these techniques more accessible to breeding programs. For instance, when the genetic architecture of a target trait is characterized using molecular methods, then artificial selection can be ameliorated through the selection of linked genes. This can be especially useful when the targeted trait is difficult to phenotype whereas the linked trait is not (Leung et al., 2020). Moreover, these methods can be useful to identify correlated responses to selection that are often driven by trade‐offs in resource allocation and gene expression. Trade‐offs can be common and have an important influence on eco‐evolutionary dynamics (Faillace et al., 2021) but they are rarely investigated in breeding programs. Finally, recent studies suggest that the exploitation of inter‐individual genetic variation can be improved with large starting populations, higher replication levels, and experimental evolution under semi‐natural environments (Bielza et al., 2020; Lirakis & Magalhães, 2019; Montserrat et al., 2021).

In contrast to the approaches described above using a species' existing gene pool, molecular methods such as genome editing and genetically modified organisms (GMOs) consist in engineering the genome of an organism in the laboratory to favor the expression of desired phenotypic traits or the generation of desired biological products. GMOs include DNA from foreign organisms into their genomes and are most often patented and thus not freely available. The majority of commercialized GMOs concerns the improvement of traits conferring resistance to insects, disease or herbicides in plants (Kos et al., 2009). For instance, the genome of *Bt* cotton contains genes from the bacteria *Bacillus thuringiensis* allowing the plant to produce an insecticide against the cotton bollworms. Recent developments in genomic approaches ease the edition of the genome of an organism and the generation of phenotypic variants by knocking‐down or knocking‐out genes (i.e., inhibiting or suppressing gene expression; Kim, 2016). The most advanced of these knockout approaches is using clustered regulatory interspaced short palindromic repeats (CRISPR) to cut DNA at specific sites (Hsu et al., 2014). This technology allows for a better understanding of gene functions and thus optimizes breeding programs. While GM plants are increasingly used, particularly in developed countries (Jacobsen et al., 2013), GM technologies have not yet been applied to BCAs and are still limited to fundamental research. Limits for the application of these technologies are their possible impacts on biodiversity as well as noncompliance with regulations of the organic food industry and policies that limit or prohibit GMO use (Gomiero et al., 2011). Although no external DNA is introduced with gene editing, this technology is often subjected to similar legal regulations as for GMOs (Alphey & Bonsall, 2018).

To conclude, human manipulations of evolution to improve biocontrol agent traits foster interest for optimizing phenotypic traits of BCAs (Leung et al., 2020; Lommen et al., 2017; Montserrat et al., 2021). Moreover, there is a heightened interest in genomic selection for complex traits with highly polygenic bases or genes with complicated epigenetic effects that are difficult to target with traditional artificial selection. Finally, recent studies have suggested to use modeling approaches to optimize artificial selection and breeding programs (Montserrat et al., 2021). For instance, Plouvier and Wajnberg (2018) proposed a modeling framework to identify key biological agent phenotypic traits from an economic perspective. This framework can be used to identify important biocontrol traits that can then be the target of breeding programs or other human manipulations of evolution schemes. Altogether, several recent reviews highlight that the exploitation of inter‐individual genetic variation and new molecular methods have the potential to improve the efficiency of biological control (Leung et al., 2020; Lommen et al., 2017; Montserrat et al., 2021). Nevertheless, the application of HME to BCAs is still in infancy and few studies artificially improved BCAs and tested their efficiency in the field. Kruitwagen et al. (2018) proposed to exploit intraspecific variations in biocontrol traits by selective breeding and illustrated their approach with parasitoids of *Drosophila suzukii*. Interestingly, a few years later, Jarrett et al. (2022) experimentally evolved two species of pupal parasitoids of *D. suzukii* and found that parasitism rate increased after only three generations. Nachappa et al. (2011) artificially selected lines of the predatory mite *Phytoseiulus persimilis* to improve their prey consumption, conversion efficiency, or dispersal. They found that the selected lines were more efficiency at controlling populations of the two‐spotted spider mite, *Tetranychus urticae*, in a heterogeneous environment. Along the same lines, Dumont et al. (2019) artificially selected zoophytophagous mullein bug *Campylomma verbasci* to generate highly zoophagous and lowly zoophagous isogroup lines. They found that, during summer, only the highly zoophagous line impacted the spider mite populations, while the lowly zoophagous line did not differ from the control treatments without BCAs. These rare examples showed that HME of BCAs can improve biocontrol. However, further studies are needed to determine which HME methods are most promising (e.g., gene editing vs artificial selection) and which traits are more likely to be optimized through HME.

## 3 NATURAL PROCESSES OF EVOLUTION AND BIOLOGICAL CONTROL

In agroecosystems, selective forces are multiple (e.g., climate, soil, agricultural practices, and multiple interacting species) and can lead to the rapid evolution of both BCAs and pests (Harmon et al., 2009). We thus need to better understand how the NPE can influence the distribution of phenotypic traits in both BCAs and pests and how this can impact biocontrol according to the biocontrol practices (i.e., classical, augmentative, or conservation biocontrol). In the next sub‐sections, we highlight the importance of NPE (i) in response to climate change, (ii) following the release of BCAs in the environment, (iii) for intra and interspecific interactions within guild of natural enemies, and (iv) in how microbial heritable symbionts harbored by pests and BCAs mediate reciprocal adaptation of these antagonistic species.

### 3.1 Evolution in response to climate change

Agroecosystems will inevitably be altered in response to climate change, suggesting a central role for the application of evolutionary principles in dealing with the consequences of these changes (Thrall et al., 2011). Climate change is associated with higher atmospheric CO_2_ concentrations, which increases mean temperatures as well as the frequency and intensity of extreme events such as drought, floods and heat waves (IPCC, 2021). Although these various abiotic drivers can influence BCAs‐pest interactions directly or indirectly through bottom‐up and top‐down effects (Han et al., 2019; Sentis et al., 2013), we here focused on the evolutionary implications of increased mean temperature and temperature extremes for the performance and the phenology of BCAs and pests. Most pests and BCAs are ectotherms and thus directly affected by temperature that impacts their performance as well as the genotypic frequencies within populations (Boukal et al., 2019; Soares et al., 2001, 2003). The effectiveness of biological control will surely depend on how fast BCAs will adapt to these environmental changes and how climate change will affect the phenotypes, distribution, and abundance of their associated pests which, in turn, can influence the evolutionary responses of BCAs to climate change (Han et al., 2019; Montserrat et al., 2021). Environmental warming may decrease top‐down control and release herbivores from predation pressure because higher trophic levels are more sensitive to climate change than lower trophic levels (Voigt et al., 2003). Alternatively, acclimation to warmer temperature can increase predator feeding rate and thus strengthen predatory impact on prey populations (Sentis, Morisson, et al., 2015). Finally, warming can disrupt host phenotypic response to BCAs and thus increase biological control efficiency. For example, winged aphids are rare under warming (Sentis et al., 2017); as a result, aphids have a reduced propensity at colonizing new plants and are subjected to strengthened top‐down control by ladybeetles (Wang et al., 2017).

The direct and indirect impact of climate change on BCAs and their pests depends on how much temperature rises relative to the thermal optimum of interacting species and on the relative thermal sensitivity of predators and prey. Warming increases predation rate up to a thermal maximum, above which predation rate decreases (Sentis et al., 2012). Thus, warming could increase predation pressure on prey in the short term, but long‐term impacts will hinge on BCA demography and the temperature‐dependent responses of their prey and host‐plants. Additionally, prolonged exposure to extreme temperatures increases mortality risk and can impact biocontrol depending on the relative impacts on predators and prey (Montserrat et al., 2013; Sentis et al., 2012, 2013). For instance, Montserrat et al. (2013) found that negative effects of high temperature are stronger for predatory mites than their prey which increases the pests' probability to escape predator control. An important challenge is to better understand how these direct and indirect effects of climate change impact the evolution of the interaction between BCAs and their pests. Unfortunately, research on these evolutionary consequences is in its infancy. Clearly, we need to identify which traits that are associated with effective biocontrol are more likely to evolve in response to climate change and to design biocontrol practices that maintain these traits at values that are beneficial for biocontrol.

Over the last decades, BCA traits associated with thermal tolerance or predation have received a growing attention to evaluate the performance of BCAs at different temperatures (Hufbauer & Roderick, 2005). For instance, there is a proliferation of studies on the thermal dependency of the functional response of BCAs with the objective of determining the range of temperatures at which they are efficient at reducing pest populations (e.g., Sentis et al., 2012; Sugawara et al., 2018). It remains unclear if the thermal sensitivity of functional response traits can evolve fast enough for BCAs to remain efficient at controlling pests with ongoing climate change although thermal acclimation can help maintaining top‐down pressure under warming (Sentis, Morisson, et al., 2015). Recent studies reported that warmer temperatures can lead to directional selection for higher thermal tolerance in herbivores following experimental evolution (Geerts et al., 2015; Harmon et al., 2009; Hoffmann & Sgro, 2011). Moreover, the evolutionary responses of BCAs to climate change could, in turn, cascade downwards to impact prey populations. For example, darker melanic morphs of the Asian ladybeetle are counter‐selected in warmer environments (Michie et al., 2010) because darker morphs have a lower thermal optimum for activity than paler morphs (Soares et al., 2003). In turn, counter‐selection of dark morphs may lead to reduced predation on aphid populations at low temperatures (de Jong & Brakefield, 1998). This suggests that biological control efficiency under climate change will depend on how fast BCAs can adapt relative to their target pests. However, there is yet too few information to conclude if the selection pressure associated with a warmer climate will be strong enough in agricultural systems to yield rapid thermal adaptation, especially for BCAs with long generation time (Faillace et al., 2021; Quintero & Wiens, 2013). Moreover, the lack of information on the genetic loci and architecture underlying thermal traits limits the development of breeding programs to improve performance under specific climate regimes.

Another impact of climate change is to advance spring and delay autumn, which lengthens the green cover period by 3–4 days per decade due (Peñuelas & Filella, 2009). These phenological changes may lead to temporal mismatches between species when they are differently affected by seasonality changes (Damien & Tougeron, 2019; Miller‐Rushing et al., 2010). They can modify host‐plant availability and quality for herbivores (Castex et al., 2018). Moreover, parasitoids are more susceptible to climatic variation than herbivores (Asch & Visser, 2007; Castex et al., 2018). In many cases, BCAs may thus lag the target pests, which would decouple ecological interactions and therefore disrupt biological control (Damien & Tougeron, 2019; Welch & Harwood, 2014). Maintaining the temporal synchronization of BCAs with their hosts may prove to be one of the most important challenges for biological control and the evolutionary potential of phenology in BCAs and pests remains to be tested. Even if adaptation to climate change occurs, this will certainly be at the cost of reduced genetic diversity, as natural selection entails loss of genetic diversity (Bush et al., 2016; Catullo et al., 2015; Eynard et al., 2016; Willi et al., 2006). This is of concern for biological control as reduced diversity is often associated with a weaker ecological impact (Des Roches et al., 2018; Raffard et al., 2019) and can compromise further adaptation due to the lack of heritable phenotypic variance.

Climate change has different evolutionary implications depending on the biocontrol practices (classical, augmentative or conservation), and it remains unclear which practice is most appropriate to cope with climate change. For classical biocontrol, the selection of BCAs performing well under native climatic conditions matching those of the area of introduction has proven successful (Mills, 2018). This good match suggests that adaptation to climate regimes is common, and therefore, if temperature increases further with climate change, choosing exotic BCAs from warmer regions could improve biological control success. For augmentative biocontrol, in regions only allowing local strains or species for biological control (Hunt et al., 2011; Lommen et al., 2017), one should exploit intraspecific genetic variation to select genotypes with optimal temperature matching to the thermal environment where the biological control is to be implemented (Lommen et al., 2017). For conservation biocontrol, it will be important to maintain large populations of natural enemies, stimulate genetic diversity, and thus increase the chances of adaptation to the warmer conditions.

### 3.2 Evolution following BCAs' release

The release of BCAs, in classical or augmentative biological control programs, may have evolutionary consequences for the populations of the target pests, the released BCAs and other nontarget species of the recipient environment (Sethuraman et al., 2020). Evolution can be rapid, driving eco‐evolutionary dynamics at contemporary scales (Sigmund & Holt, 2021) and, therefore, considering the evolution of organisms in novel environments is essential for classical or augmentative biological control programs (Wepprich & Grevstad, 2021).

The success of establishment and spread of an introduced species may originate from a combination of pre‐adapted traits of ecological importance, their plasticity, their rapid evolutionary changes after introduction due to adaptive response to natural selection and stochastic changes resulting from introduction history as well as founder effects and genetic drift (Castillo et al., 2021; Colautti & Lau, 2015). It is expected that exotic species would be more evolutionary naïve in the new environment than native species used in augmentative biocontrol projects. The success of exotic BCAs compared with indigenous ones will thus depend more on the subsequent evolutionary response to selection (Wu & Colautti, 2022).

We will here discuss three main points driving the success of establishment and spread of the introduced species: (i) the importance of the genetic diversity and plasticity of the BCA founder population, (ii) the issue of enemy release of BCAs in the recipient environment, and (iii) how to use evolutionary information to prevent the side effects of introduced BCAs on nontarget hosts. Interestingly, the introduction of BCAs in classical or augmentative biological control programs can be equated to planned invasions (Abram & Moffat, 2018; Blackburn et al., 2011; Ehler, 1998; Mills, 2018). Consequently, knowledge on the evolution of invasive species and the communities of organisms of the invaded environment can contribute to inform biological control strategies, as in some of the examples we visit below.

**FIGURE 1:**
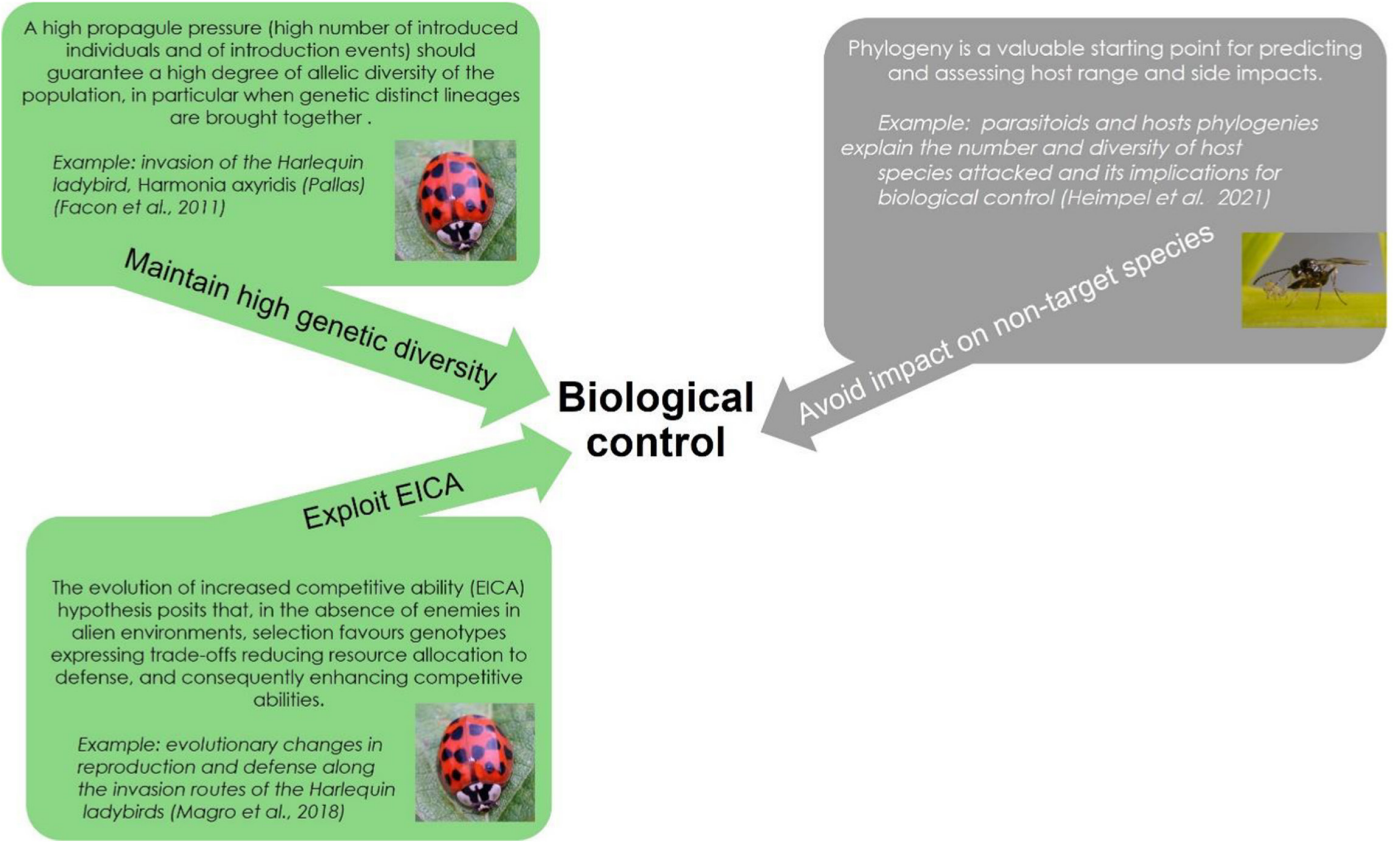
Three evolutionary aspects to be considered when releasing a BCA to a new environment. (i) Establishment of natural enemies is better if their genetic diversity is high because small founder populations are subjected to the Allee effect, genetic drift and inbreeding that frequently result in the fixation of deleterious alleles and a reduced capacity for local adaptation. (ii) Enemy release in new habitats benefits natural enemies both directly through lower mortality and indirectly through the selection of genotypes with low resource allocation to defense and high allocation to competitive abilities. (iii) Host range of natural enemies is liable to evolve depending on the environment, and phylogenetic approaches are useful to predict the likelihood of impacts of biological control on nontarget species (e.g., parasitoids and their hosts, Heimpel et al., 2021).

The success of establishment, expansion, and impact of a population after release is primarily driven by the genetic diversity of key phenotypic traits of the founder population (Figure 1, Lambrinos, 2004). Small populations are prone to genetic drift and inbreeding, which can result in the fixation of deleterious alleles and a reduced capacity for local adaptation. A high propagule pressure, that is, high number of introduced individuals and introduction events, in particular when genetic distinct lineages are brought together, should guarantee a high degree of allelic diversity of the population. The Harlequin ladybird *Harmonia axyridis* native from Asia was first introduced as a BCA in the USA where it has since become invasive, as in several other countries around the world (Roy et al., 2016). The admixture of the populations of *H. axyridis* contributed to its spreading and establishment success through the selection of three main traits: body size, shorter development time, and increased ability to resist starvation (Facon et al., 2011). Hemptinne et al. (2012) showed that body mass is positively correlated with reproductive rate and movement speed and is, therefore, a good predictor of the ability to spread and colonize new territories, while also being advantageous life history traits for a biological control agent (Heimpel & Mills, 2017). Unfortunately, the parallel adverse ecological impact of *H. axyridis*, particularly on the biodiversity of the recipient ecosystems, now prevents its use in biological control programs.

The absence of natural enemies has consequences for the establishment and spread of the released BCAs. Enemy release benefits the newcomer directly through decreased mortality rate but might also be advantageous indirectly. Blossey and Notzold (1995) postulate that, under enemy release, NPE can favor genotypes with low resource allocation to defense and high allocation to competitive abilities, and this has been tested for several organisms (Atwood & Meyerson, 2011; Blossey & Notzold, 1995). Indeed, resources invested in defense are traded off against growth and reproduction in plants (Herms & Mattson, 1992) and in animals (Braendle et al., 2011). For BCAs, Magro et al. (2018) reported evolutionary changes leading to an increase in reproduction and a decrease in defense for three populations of the ladybird *H. axyridis* along its invasion route (Figure 1). This and other studies also show that, at length, natural enemies in the introduced range might adapt to the exotic BCA leading to a shift in selection toward better defended genotypes with negative consequences for competition capacities (Knapp et al., 2019), which could eventually limit the BCA success in biological control programs.

In biological control projects, prey or host specificity is a major concern. Gougherty and Davies (2021) defend that invasive herbivores are more likely to establish and spread on plants that are phylogenetically closely related to their native plant hosts and suggest that phylogenetic conservatism in prey/host preferences can similarly inform the selection of BCAs. Indeed, it has long been recognized that phylogenetic relatedness can be a proxy for ecological similarity and a useful way to predict ecological interactions, although this should not be taken as an absolute rule (Losos, 2008). In this context, the Organisation for Economic Co‐operation and Development (OECD) recommends that during host specificity testing, available information on phylogenetically related nontarget hosts should be one of the elements to be taken into consideration (Babendreier et al., 2006). As a matter of fact, phylogeny is a valuable starting point for predicting and assessing host range (Kuhlmann et al., 2006). For instance, Heimpel et al. (2021) investigated how parasitoid and host species phylogenies can influence the number and diversity of host species attacked by parasitoids and its implications to biological control (Figure 1). Unfortunately, arthropod phylogeny lags well behind plants (Kuhlmann et al., 2006; Regier et al., 2010) which limits the prediction on host range in BCAs.

### 3.3 Evolution of intra and interspecific interactions within guild of natural enemies

Conservation biological control mainly consists in enhancing natural enemy abundance, diversity, and functional efficiency through agricultural practices (e.g., providing to natural enemies food sources or refugia; Settele & Settle, 2018). At first glance, it may be suggested that increasing BCA diversity has positive effects on pest control: by favoring various BCAs, a wider range of pest species is affected, and pest populations are exposed to a diversity of selective pressures, reducing their adaptive response. However, several studies have shown that favoring BCA diversity and abundance may not lead to decreased pest damage (Jonsson et al., 2008). One reason for this is probably a poor understanding of intra‐ and interspecific interactions within natural enemies guilds (e.g., Straub et al., 2008). Several studies have reported that the effect of increased natural enemy diversity on biological control may range from negative to positive (Holt & Polis, 1997; Sentis et al., 2014). For instance, intraguild predation among coexisting natural enemies can dampen the strength of trophic cascade and reduce the efficiency of conservation biological control strategies (Cloyd, 2020). The risk of intraguild predation may lead to avoidance adaptations in some BCAs (e.g., Nakashima et al., 2004). In contrast, positive effects of BCA diversity on biological control primarily occur when the feeding niches of the BCAs complement each other (Schmitz, 2007; Ximenez‐Embun et al., 2014). In agreement with this, Northfield et al. (2010) showed that a guild of natural enemies of aphids partition resources according to their microhabitat and the relative strength of their intra and interspecific interactions. This suggests that ecological niche theory can be used to design optimal assemblages of BCAs. Nevertheless, ecological niches can evolve in nature, and the evolution of ecological niches of BCAs is driven by selective factors that are external or internal to their community (Bolnick et al., 2011; Outreman et al., 2018; Raffard et al., 2021; Roff, 1997; Violle et al., 2012). The environmental filtering theory suggests that local environmental conditions (i.e., external filters) should induce phenotypic convergence at the community level. Such phenotypic convergence is then expected when regional environmental conditions exert strong pressure on species (Weiher et al., 2011) and leads to a decline in the trait values range among coexisting individuals (e.g., Enquist et al., 2015). Inversely, in competitive contexts (i.e., internal filters), species belonging to a community may present contrasting traits as a means of niche differentiation, allowing coexistence (Losos, 2008; MacArthur & Levins, 1967). By studying a guild of aphid parasitoids, Outreman et al. (2018) showed that the patterns of trait variation across various French regions are consistent with local adaptation while the patterns of phenotypic variation within regions suggested how coexistence modulates life history traits expression through niche differentiation. A central challenge is to disentangle the selective filters that drive local patterns of phenotype variation within BCA communities and application of trait‐based studies has the potential to provide further insight into the main role of agricultural practices on BCAs' efficiency.

A better knowledge of how intraspecific variation and evolution modulate trophic interactions could help improving biological control as shown by the following examples. Most organisms have evolved sophisticated chemical sensory systems to detect subtle variations in chemical information (Cardé & Millar, 2004; Peñuelas & Staudt, 2010). The evolution of chemical sensing thereby strongly determines microhabitat use and thus the extent of both intra and interspecific interactions (Mondor et al., 2004; Sentis, Ramon‐Portugal, et al., 2015; Yuan et al., 2009). For instance, like many other invertebrates, several predators and parasitoids of aphids rely on infochemicals related to brood to assess the presence of conspecific and heterospecific individuals (Blomquist & Bagnères, 2010; Magro et al., 2007). By doing so, they avoid foraging and laying eggs in colonies already visited and thus decrease the risk of competition, cannibalism, and/or intraguild predation to their offspring (Doumbia et al., 1998; Nakashima et al., 2006; Raymond et al., 2000). Interestingly, aphidophagous ladybirds tend to avoid tracks of ladybird species with which they coevolved but react less to species from which they are further away in the phylogenetic tree (Magro et al., 2007, 2010, 2017). Intraspecific and interspecific avoidance generates resource partitioning because areas on plants that were already visited by conspecifics are avoided by subsequent individuals (Gil et al., 2004). As a result of resource partitioning, diverse communities extract more resources from their environment than less diverse ones (Cardinale et al., 2006).

Intraspecific effects on ecological dynamics and ecological processes are often comparable to, and sometimes stronger than, species effects (Des Roches et al., 2018; Raffard et al., 2019). For instance, intraspecific variation in niche exploitation can be important for the occurrence and strength of trophic cascades—the indirect effect of predators on nonadjacent lower trophic levels. In a recent study, Sentis et al. (2020) experimentally investigated how herbivore intraspecific genetic variation and evolutionary divergence related to host‐plant specialization influences trophic cascade strength in a ladybeetle‐aphid‐broad bean system. They found that the occurrence and strength of the trophic cascade strongly depend on herbivores' intraspecific variation and evolutionary divergence associated with host‐plant specialization. Along the same line, animal personality, defined as consistent individual differences in behaviors, can be important for top‐down control and biological control. For instance, Start and Gilbert (2017) conducted a mesocosm experiment with aquatic food webs and found that the more active dragonfly nymphs disproportionately reduce the abundance of prey and induce stronger trophic cascades than less active individuals. These results suggest that intraspecific variation in predator personality is an important determinant of prey abundance, community composition, and trophic cascades. Altogether, these findings stress the importance of intraspecific trait diversity and evolutionary adaptations as drivers of top‐down control and underline that intraspecific variation should not be overlooked in biological control programs.

### 3.4 Symbiotic microbes and the evolution of pests–natural enemies' interactions

The endosymbiotic microorganisms living in the body of animals can have profound effects on their phenotypes (McFall‐Ngai et al., 2013) and could thus play an important role in pests and BCAs evolutionary responses to ecological factors with consequences for biological control efficiency. In this section, we highlight the roles of symbiotic microbes on the evolution of pests and BCAs and their antagonistic coevolution.

**FIGURE 2:**
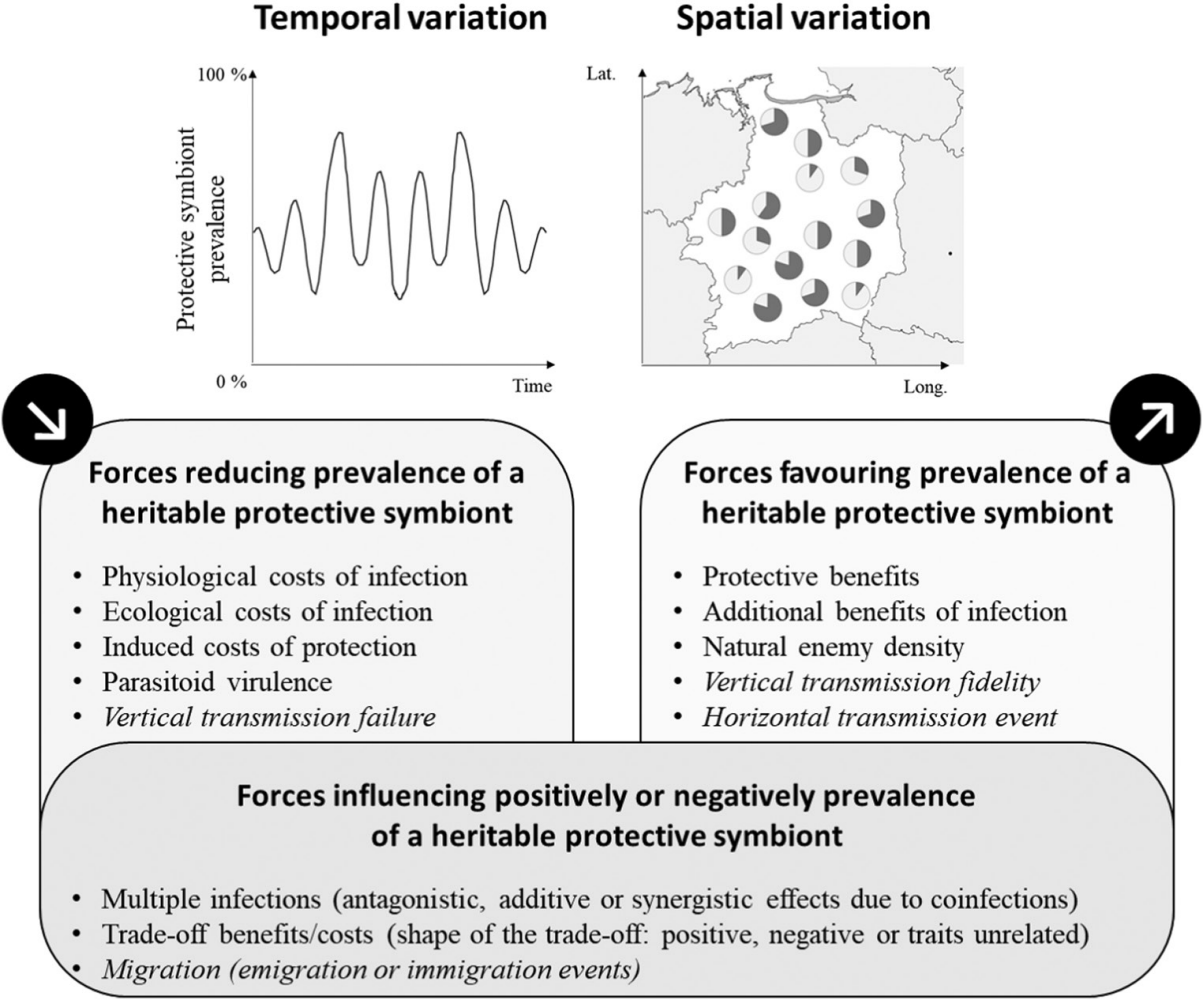
Selective and nonselective (italic text) forces influencing frequencies of protective heritable symbionts in host natural populations. These forces can explain temporal and spatial variations of protective symbionts in nature and are modulated by the genomes of the interacting species and/or local environmental conditions.

If diversity of microbial symbionts has been determined in an increasing number of notorious pests (e.g., lepidopterans (Mereghetti et al., 2017), fruit flies (Broderick & Lemaitre, 2012; Noman et al., 2020), bark beetles (Chakraborty et al., 2020), and aphids (Gauthier et al., 2015; Ma et al., 2021)), their role on pest evolution has been explored particularly on few taxa such as stinkbugs (Sudakaran et al., 2015), aphids (Oliver et al., 2010), fruit flies (Ballinger & Perlman, 2019), and spider mites (Rodrigues et al., 2022). Depending notably on the transmission routes of symbionts, microbial associations may be parasitic or mutualistic: while horizontal transmission is prone to the evolution of selfish strategies, vertical transmission often favors mutualistic interactions (Frank, 1996). Among mutualistic heritable associations, most of the best‐described are based on defensive services provided by symbionts to hosts (Berasategui et al., 2016): some microbial symbionts confer a protection to their host against natural enemies (Oliver et al., 2014). Protective symbioses have been extensively studied in aphids (see Table S1 for a list of aphid symbionts), where some bacterial associates can confer resistance against pathogens (Heyworth & Ferrari, 2015; Scarborough et al., 2005), predators (Polin et al., 2015), and parasitoids (Oliver et al., 2003; Vorburger et al., 2010). The protection against parasitoids mainly implies *Hamiltonella defensa*, a bacterium that can confer resistance in different aphid species (Oliver et al., 2003, 2005; Schmid et al., 2012; Vorburger et al., 2010), through the presence of a toxin‐encoded by a bacteriophage (Degnan & Moran, 2008). The strength of the *H. defensa*‐mediated protection varies from nil to complete and mainly depends on the genomes of all interacting players (i.e., bacteriophage‐bacterium‐aphid‐parasitoid genomes; Cayetano et al., 2015; Leclair et al., 2016). Interestingly, *H. defensa* infection induces fitness declines for aphid hosts, either directly through physiological costs (Oliver et al., 2008; Simon et al., 2011) or indirectly through ecological costs (i.e., higher vulnerability to predators; Dion et al., 2011; Polin et al., 2014). Fitness costs induced by the protection deployment under parasitism challenge have also been noted (Vorburger et al., 2013). Like the protection, fitness costs vary depending on interacting players' genome and local conditions (Cayetano et al., 2015; Leclair et al., 2016; Sochard et al., 2019). The benefit/cost balance of symbiont infection plays an essential role in the selection of pest‐microbe‐associated genomes in nature (Cayetano et al., 2015) and maintenance of resistance. For example, Oliver et al. (2008) showed that frequencies of aphids harboring protective microbes increase rapidly under wasp parasitism challenge while they decline under low risk of parasitism. Figure 2 details selective and nonselective forces impacting frequencies of protective symbionts in natural populations.

As protective symbioses obviously affect the performance of natural enemies, they also mediate their evolution. Vorburger and Perlman (2018) analyzed the impacts of such symbioses on parasite virulence evolution and identified (i) under which conditions the protective symbiont may take over from the host the reciprocal adaptation with parasitoids and undergo its own selection dynamics, thereby altering or relaxing selection on the hosts' own immune resistance and (ii) the underlying mechanisms of the protection resulting in the evolution of parasitoid virulence. Monticelli et al. (2019) reviewed how protective symbionts found in pests influence parasitoid foraging strategies, with consequences on host exploitation and parasitoid fitness, ultimately mediating the evolution of host range. Various studies found that symbionts could narrow parasitoid host range (Frago et al., 2017; Kraft et al., 2017; McLean & Godfray, 2017; Nyabuga et al., 2010) and such symbiotic effects may produce divergent selection associated with the use of particular hosts, resulting in localized genetic structuring of parasite populations that may impede or restrict an expansion in host range (Henry et al., 2008). From all these evolutionary effects on parasitoid individuals, protective symbionts can also change parasitoid communities: the presence of protective symbionts in a pest community can led to variation in pest densities and parasitoid diversity, modulating species networks (Sanders et al., 2016).

The presence of protective symbioses in pest species must be obviously accounted for in the development of biocontrol programs (Vorburger, 2018): the production of BCAs and their release on targeted cultures should integrate all forces modulating symbiotic protection. Two experimental evolution studies showed that a large genetic variation in parasitoid populations is needed to overcome symbiotic protection of pests (Dion et al., 2011; Rouchet & Vorburger, 2014). As protective symbioses depend on complex genotype per genotype interactions among hosts, symbionts and parasitoids (e.g., Schmid et al., 2012), various protective symbiont strains might be offered to the BCA populations and high genetic variability in BCAs is needed to control pests. A large initial population of pests and parasitoids, with various field origins and regular supplementation, is needed for BCA mass rearing. Concerning BCA application, Vorburger (2018) recommends to release high densities of parasitoids as successive parasitoid attacks can reduce the survival of protected individuals.

So far, microbial associates harbored by BCAs received scarce attention and have been mainly studied in parasitoids (reviewed in Dicke et al., 2020). In contrast, a wide variety of viruses has been reported as symbionts of parasitic wasps (Beckage & Drezen, 2011; Engelstädter & Hurst, 2009), the majority being polydnaviruses (PDVs; Dicke et al., 2020). Some of these PDVs can enhance parasitoid virulence by suppressing parasitoid's host immune response (Strand & Burke, 2019). Also, the presence of PDVs in the parasitoid's host may affect its traits as well as plant responses to herbivory and subsequently alter plant interactions with other organisms (Cusumano et al., 2018; Tan et al., 2018; Zhu et al., 2018). Due to the incidence of PDVs on both pest immunity and tritrophic interactions, these viral symbionts open the way to new practices in biocontrol (Cusumano & Volkoff, 2021). By generating genetically transformed plants expressing PDVs genes, the PDVs proteins are used as bioinsecticides against pest caterpillars (Di Lelio et al., 2014; Gill et al., 2006). Another way to use PDVs in biocontrol is to include the gene of interest in the genome of an entomopathogenic virus (Wei et al., 2016). Also, studying how PDVs affect herbivore traits and, by extent, parasitoid host range, could be used to produce adapted parasitoids. Like protective symbionts in pests, if viral symbionts in parasitoids influence host quality and parasitoid performance, then it is worth including viral genetic variation in BCAs selection processes.

To conclude, symbionts of both pests and BCAs may have profound effects on the efficiency of biological control. Their importance for the evolution of BCA traits is still largely unexplored but could play an important role in natural evolution and offer new perspectives for human‐manipulated evolution.

## 4 ON THE INTERPLAY BETWEEN HUMAN‐MANIPULATED AND NATURAL EVOLUTION

While HME and NPE can both be important for biological control, they differ in their levels of control and finality. HME mostly happens under controlled environments and targets specific traits of interest for biological control. In contrast, NPE is less predictable as it results from the diversity of selective pressures and how these pressures and stochastic processes influence trait distribution and the dynamics of phenotypes (Table 1). Ultimately, artificially improved BCAs are used to control pests in agricultural or natural systems where selection pressures are multiple and more diffuse. It is thus important to consider the interplay between human‐manipulated and natural evolution when designing breeding programs or other genomic approaches to improve BCAs' traits. One iconic example of this interplay is the case of a breeding program for the Asian ladybeetle *H. axyridis*. The aim was the selection of wingless adults to reduce female mobility and thus increase their numerical response on plants infested by aphids (Lommen et al., 2017; Tourniaire et al., 2000). The breeding program successfully resulted in wingless adults, but the field efficiency of the selected strain was lower than expected because natural selection favors females that lay a single batch of eggs and then leave the plant to avoid intraspecific competition for prey. If they land on a plant already occupied by conspecific, they also withhold from egg laying and move away (Fréchette et al., 2003; Gil et al., 2004; Hemptinne & Magro, 2015; Sentis, Ramon‐Portugal, et al., 2015). Consequently, the selected wingless adults were walking away from oviposition sites already occupied by conspecifics. Moreover, the wingless trait is recessive and can be lost in a single round of interbreeding with normal (flight capable) individuals (Riddick, 2017) and it remains difficult to establish the benefits of this wingless strain for biocontrol (Lommen et al., 2013). This example highlights the importance of considering the NPE when designing breeding programs or other human‐manipulated evolution. We need to understand how NPE shapes the life history strategies of BCAs and to maintain laboratory populations of BCAs that are as much as possible representative of natural populations and that harbor sufficient genetic diversity. This can be achieved by mixing the genetic background of several field populations via controlled crosses when creating laboratory populations. Using such outbred populations allows maintaining high genetic variability and thus limits the influence of genetic bottlenecks and other stochastic processes. Moreover, it is then possible to generate inbred lines, allowing each genotype to be studied independently to better understand the phenotypic and genotypic variability for relevant traits (Godinho et al., 2020).

Recent studies suggest that the artificial exploitation of inter‐individual genetic variation can be improved with experimental evolution under semi‐natural environments (Bielza et al., 2020; Godinho et al., 2020; Lirakis & Magalhães, 2019; Montserrat et al., 2021). This would be a powerful way to avoid disconnecting HME from NPE while increasing the likelihood that the target traits are being selected and maintained. Moreover, molecular tools can provide a better understanding on how artificially improved BCAs evolve both during their artificial improvement and once they are released in agroecosystems. For instance, genetic markers (e.g., microsatellite, SNPs) are used to monitor genetic variation either to avoid the loss of genetic variation and genetic inbreeding during mass rearing of BCAs and to track genetic variation, field performance, and ecological risk of agents released into the field. Furthermore, whole‐genome genotype‐by‐sequencing (GBS) can allow estimation of allele frequency to track evolution at the genomic scale, identify regions under selection, and gather information on the genetic architecture of traits of interest (Davey & Blaxter, 2010). These technological advances should thus provide a better understanding on how BCA traits selected by HME are affected by the natural processes of evolution after BCA release in agroecosystems.

Human manipulations of evolution was paramount in the domestication of plants, animals and microorganisms as well as with recent technological advances such as gene editing, and GMOs (Kos et al., 2009). Nevertheless, the lesson from the Anthropocene is that human‐designed systems can have extremely powerful detrimental effects that threaten global health. It remains unclear if we can assess the secondary effects of BCAs produced by human‐induced evolution on biodiversity and ecosystems. For instance, plant GMOs impact nontarget arthropods (Pilcher et al., 2005), and herbicide‐resistant crops already have significant evolutionary consequences on insects (Zalucki & Lammers, 2010) and cause pesticide resistance evolution in weeds (Duke & Powles, 2008; Powles & Preston, 2006). Understanding the intricacies of evolution in agricultural or natural systems remains a challenge, but anticipating the evolutionary consequences of releasing organisms manipulated in the laboratory might be even more challenging (Powles, 2008).

Modern agriculture is mainly designed to maximize primary production and not to trade off primary production with low level of pest damages. Therefore, intensive agriculture is continuously applying strong selection pressures on pests, which in turn become more tailored to it. For example, the Northern and Western corn rootworms (*Diabrotica barberi* and *D. virgifera*) overcame annual crop rotations between corn and soya beans, by developing the ability to diapause at least 2 years (Turcotte et al., 2017). These two pests are now perfectly synchronized with their host‐plant, which probably maximize their fitness. In England, in the last 15 years, an aphid clone of *Sitobion avenae*, that evolved resistance to pyrethroid insecticides used in cereal fields, expanded its geographic range, and increased in proportion in populations from South to North (Morales‐Hojas et al., 2020). Such a large‐scale geographic homogenization of populations is also observed with *Bemisia tabaci* in the USA (Gautam et al., 2020). These examples show how intensive agriculture can have negative effect on pest control when it leads to their rapid evolution. In contrast, natural enemies displaying specific agricultural adaptations are rare (Turcotte et al., 2017; but see Nouhuys & Via, 1999), which may be explained by their lower fecundity, longer lifespan, and generation time that limit their adaptability or by the lack of studies addressing this topic. If further studies confirm that agriculture intensification modifies herbivore traits more frequently or faster than BCA traits, then pests may benefit from an enemy‐free space provided by intensive agriculture.

The former examples of rapid evolution in pests indicate that greater emphasis should be placed on agroecosystem redesign to counter evolutionary forces favoring pest outbreaks. For example, organic farming favors higher species diversity (Lichtenberg et al., 2017) as do agricultural landscapes harboring a proportion of 20% natural habitat, farms applying crop rotations longer than 5 years, having narrow rectangular fields of a maximum of 6 ha the width of which should be compatible with agricultural machinery (Tscharntke et al., 2021). These agricultural settings favor a high level of conservation biocontrol. It would be interesting to test whether such agrosystems are able to maintain a high regional genetic diversity of species of BCAs and thus favor evolutionary processes for the adaptation to rapid environmental changes. By promoting genetic diversity, conservation biocontrol could thus offer a way to promote NPE and thus increase the chances that BCAs adapt to their local environments.

## 5 CONCLUSION AND FUTURE DIRECTIONS

**FIGURE 3:**
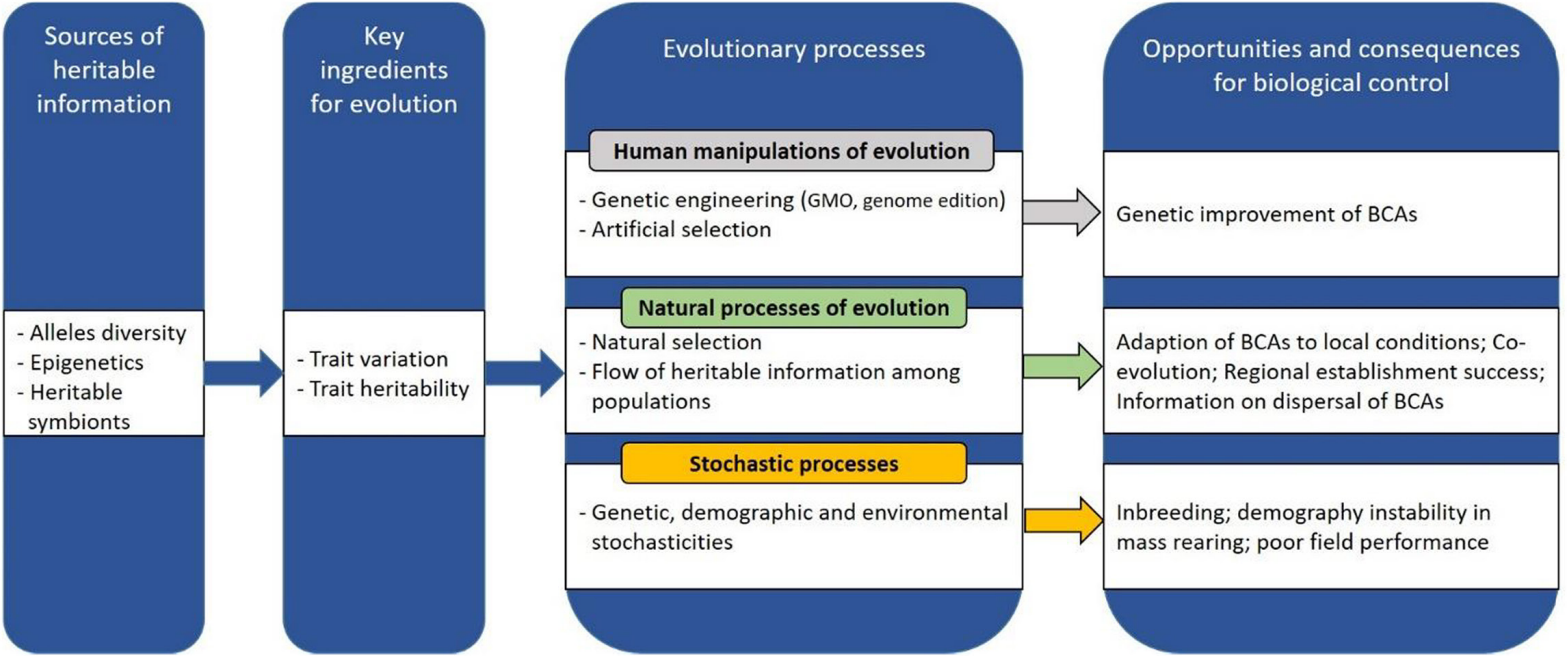
Key components for trait evolution, evolutionary processes, and consequences for biocontrol. A source of heritable information (genes, epigenetics, or heritable symbionts) and inter‐individual trait variation are required for trait evolution. Three main types of evolutionary processes (human manipulations of evolution, natural processes of evolution, and stochastic processes) can lead to trait evolution. Selection, inbreeding, drift, and flow of heritable information (e.g., gene flow) among populations together shape the structure or adaptive and neutral heritable variation within and among populations. In the case of human manipulations of evolution, strong and directional selective forces can lead to rapid genetic improvements of BCA traits of interest for biocontrol such as mass rearing, attack rate, and thermal tolerance. Depending on environmental stochasticity, population size, and diversity, stochastic processes can induce genetic drift and inbreeding, which can lead to issues with mass rearing and field performance.

Biological control can help achieve resilience and sustainability in agricultural systems. We thus need to better understand key ecological and evolutionary processes modulating its success. Evolution processes are likely to become increasingly important for biological control given the need (i) to find ways to improve the efficiency of local BCAs and (ii) to integrate the role of global change on both pests' and BCAs' evolution. There is thus a growing interest for the use of evolutionary and genetic approaches to optimize traits of interest (Leung et al., 2020; Lommen et al., 2017; Montserrat et al., 2021; Wajnberg, 2004). This effervescence translates into the development of new approaches for the optimization of phenotypic traits such as genomic selection, microbiota optimization, and modeling approaches. These new developments should help classical or augmentative biological control but may be more limited in the case of conservation biological control where selection pressures are more diffuse and unpredictable, and where the community context often plays a more important role (Figure 3). In such case, knowledge on the evolutionary potential of BCAs and how this influences their intra and interspecific interactions is helpful to achieve more predictive biological control programs. Recent progress in investigating the microbial community associated with major pests (e.g., bacterial associated) and control agents (e.g., viral associated) offers new possibilities of improving biological control by targeting symbionts, studying their incidence on evolution of their hosts, and then developing strategies to use symbiont(s) to control insect pests. Finally, promising new developments such as the use of modeling frameworks (Plouvier & Wajnberg, 2018) or food web engineering (i.e., the combination of evolutionary biology and food web theory to engineer webs maximizing pest control and trophic cascade strength; Montserrat et al., 2021) are emerging and should pave the way for a better integration of evolutionary approaches into biological control programs. Altogether, the combination of these different approaches and information helps understanding the consequences of rapid environmental change for biological control and optimize BCAs to remain efficient under a changing range of environmental conditions.

Despite recent advances in integrating evolutionary approaches in biological control, there are still important challenges to be addressed to achieve efficient evolutionary biological control. First, the transfer of academic research and results toward the biological control industry, farmers, and practitioners should be dramatically improved by at least including evolutionary biology in agricultural teaching programs. Second, there are very few long‐term studies on biological control and the evolutionary potential of BCAs (most studies are short‐term laboratory or semi‐laboratory experiments; Montserrat et al., 2021). Long‐term studies are needed, especially in the case of conservation biological control, where the role of evolution is less well characterized. Third, more research on micro‐BCAs (viruses, bacteria, and fungi) is required because of their high evolutionary potential associated with their short generation time and high population abundance.

## CONFLICT OF INTEREST

The authors have no conflicts of interest to declare.

## Supporting information


Table S1

## Data Availability

This study does not include data.
