# Supplementary material for: Biological control needs evolutionary perspectives of ecological interactions
Source: Evol Appl. 2022 Nov 1;15(10):1537–54. doi: 10.1111/eva.13457 (PMC9624075; doi:10.1111/eva.13457)
Supplement: Supplementary file 1 — Table S1 [file EVA-15-1537-s001.docx]

**Supplementary information**

**Biological control needs evolutionary perspectives of ecological interactions**

Table S1. Examples of protection against natural enemies that aphids derived from symbionts

| Aphid | Symbiont | Protection | Reference |
| --- | --- | --- | --- |
| ***Myzus persicae*** | *Regiella insecticola* | Against parasitoids | Vorburger et al., 2010)- |
| ***Acyrthosiphon pisum*** | *Fukatsuia symbiotica*  *Serratia symbiotica* | Against parasitoids | Guay et al., 2009  Leclair et al., 2016 |
|  | *R. insecticola*  *Rickettsia viridis*  *Rickettsia sp.*  *Spiroplasma sp.*  *F. symbiotica* | Against fungal pathogen | Heyworth & Ferrari, 2015  Łukasik et al., 2013 Scarborough, 2005 |
|  | R. viridis | Against predator | Polin et al., 2015 |
